# High-magnitude compression accelerates the premature senescence of nucleus pulposus cells via the p38 MAPK-ROS pathway

**DOI:** 10.1186/s13075-017-1384-z

**Published:** 2017-09-18

**Authors:** Pei Li, Gang Hou, Ruijie Zhang, Yibo Gan, Yuan Xu, Lei Song, Qiang Zhou

**Affiliations:** 1 Department of Orthopaedic Surgery, No. 89 hospital of PLA, Weifang, Shandong 261026 China; 2 Department of Orthopaedic Surgery, Southwest Hospital, Third Military Medical University, Chongqing, 400038 China; 3 Department of Orthopaedics, Third Affiliated Hospital of Sun Yat-sen University, Guangzhou, 510700 China; 4 Department of Respiratory Medicine, the Third Xiangya Hospital, Central South University, Changsha, Hunan 410013 China; 5 Department of Orthopaedic Surgery, Xinqiao Hospital, Third Military Medical University, Chongqing, 400037 China

**Keywords:** Compression, Senescence, Nucleus pulposus cell, p38 MAPK, Reactive oxygen species

## Abstract

**Background:**

Mechanical overloading can lead to disc degeneration. Nucleus pulposus (NP) cell senescence is aggravated within the degenerated disc. This study was designed to investigate the effects of high compression on NP cell senescence and the underlying molecular mechanism of this process.

**Methods:**

Rat NP cells seeded in decalcified bone matrix were subjected to non-compression (control) or compression (2% or 20% deformation, 1.0 Hz, 6 hours/day). The reactive oxygen species (ROS) scavenger N-acetylcysteine (NAC) and the p38 MAPK inhibitor SB203580 were used to investigate the roles of the ROS and p38 MAPK pathway under high-magnitude compression. Additionally, we studied the effects of compression (0.1 or 1.3 MPa, 1.0 Hz, 6 hours/day) in a rat disc organ culture.

**Results:**

Both in scaffold and organ cultures, high-magnitude compression (20% deformation or 1.3 MPa) increased senescence-associated β-galactosidase (SA-β-Gal) activity, senescence marker (p16 and p53) expression, G1 cell cycle arrest, and ROS generation, and decreased cell proliferation, telomerase activity and matrix (aggrecan and collagen II) synthesis. Further analysis of the 20% deformation group showed that NAC inhibited NP cell senescence but had no obvious effect on phospho-p38 MAPK expression and that SB203580 significantly attenuated ROS generation and NP cell senescence.

**Conclusions:**

High-magnitude compression can accelerate NP cell senescence through the p38 MAPK-ROS pathway.

**Electronic supplementary material:**

The online version of this article (doi:10.1186/s13075-017-1384-z) contains supplementary material, which is available to authorized users.

## Background

Intervertebral disc degeneration (IDD) is a leading cause of low back pain and leads to a heavy socioeconomic burden on the health care system. Cellular senescence accumulation within the disc tissue is common during disc ageing and degeneration [1–3]. Notwithstanding this important discovery, the potential mechanism of disc cellular senescence is not fully understood.

Since ageing is one of the main contributors to IDD, the natural ageing pathological process may lead to the cellular senescence during IDD [4]. A previous study demonstrated age-dependent disc cell senescence in human disc samples [5]. However, several studies [6–8] did not find an obvious correlation between the donor’s age and cellular senescence accumulation. Correspondingly, the senescence-related markers do not show an age-related upregulation in degenerative discs [6, 7, 9]. These findings indirectly suggest that there may be some other factors that can aggravate cellular senescence during disc degeneration and disc ageing apart from the natural ageing process.

In vivo, the intervertebral disc (IVD) is subjected to numerous physiological and non-physiological mechanical loads. It is generally accepted that mechanical load plays an important role in the process of IDD and that a non-physiological load may initiate and accelerate disc degeneration [10–12]. Additionally, a previous study on rat disc degeneration model induced by foreleg amputation showed increased senescent cells within the degenerative disc [13–15]. The forelimb amputation will cause the experimental animal to experience prolonged upright postures and thus increased axial compression [16], indicating that overloaded compression may contribute to increased cellular senescence within the disc. It is well established that nucleus pulposus (NP) cells are responsible for matrix biosynthesis within the NP region, which first exhibits degenerative changes during disc degeneration. However, as far as we know, no direct comprehensive evidence about the effects of compression on NP cell senescence and the underlying molecular mechanism behind this process can be found.

Based on the free-radical theory of ageing, oxidative stress caused by reactive oxygen species (ROS) contributes to cellular function decline, which is involved in cellular senescence [17]. Numerous disorders, including osteoarthritis and neurodegenerative disease, are reported to be related to oxidative stress [18, 19]. In degenerative human discs, ROS generation is also elevated [20–22] and the accumulated ROS are reported to be involved in age-related disc degeneration [22, 23]. Importantly, excessive mechanical loading is reported to increase the release of ROS from mitochondria in cartilage [24–27]. Mitogen-activated protein kinases (MAPKs) are part of the key pathway for the transduction of extracellular stimuli into cellular biological responses [28]. Previous studies demonstrated that the p38 MAPK signalling pathway can be activated by mechanical stimulation [29–31] or ROS accumulation [32]. We postulate that the p38 MAPK pathway and ROS may modulate the effects of mechanical stimulation on NP cell biology.

In the present study, we mainly aimed to investigate whether high-magnitude compression can accelerate NP cell senescence and the molecular mechanism behind this process using the NP cell scaffold culture and the intact rat disc organ culture. NP cell senescence was evaluated from direct and indirect parameters including cell proliferation, telomerase activity, G1 cell cycle arrest, senescence associated β-galactosidase (SA-β-Gal) activity, glycosaminoglycan (GAG) content, matrix macromolecule (aggrecan and collagen II) expression and senescence marker (p16 and p53) expression. The ROS scavenger N-acetylcysteine (NAC) and the p38 MAPK inhibitor SB203580 were used to investigate the role of ROS and p38 MAPK pathway in this regulatory process.

## Methods

### Ethical statement

The experimental animals used in the present study were obtained from the Animal Center of Third Military Medical University. All approved animal experiments were performed in accordance with relevant guidelines and regulations of the Ethics Committee at Southwest Hospital affiliated to the Third Military Medical University (SYXK (YU) 2012-0012).

### Investigation of the effects of high-magnitude compression on the premature senescence of NP cells in rat NP cell scaffold culture

#### Isolation and scaffold culture of rat NP cells

NP cells from 35 Sprague-Dawley rats (male, 250 g and 6–8 weeks old) were isolated. Briefly, after animals were sacrificed with carbon dioxide, the thoracic and lumbar discs were separated, the soft tissues were removed, and the innermost NP tissue was separated under a dissecting microscope. Then, sequential enzymatic digestion with 0.25% trypsin for 5–7 minutes and 0.25% Type I collagenase (Sigma-Aldrich, St Louis, MO, USA) for 10–15 minutes at 37 °C was performed. Thereafter, the collected NP cell pellets were re-suspended in a monolayer culture with DMEM/F12 (HyClone, Logan, UT, USA) that contained 10% (v/v) foetal bovine serum (FBS, Gibco, Carlsbad, CA, USA) and 1% (v/v) penicillin-streptomycin (Gibco, USA). NP cell phenotype was identified by morphology and NP cell specific marker (CAXII, Keratin-19, FOXF1 and PAX1) expression (Additional file 1: Figure S1) [33]. To avoid the influence of cell passage on cellular senescence [34], passage 2 (P2) NP cells suspended in the collagen solution (1 mg/mL, Shengyou Biotechnology Co., Ltd., Hangzhou, China) were seeded in the prepared bovine decalcified bone matrix (DBM, 10 × 10 × 5 mm, 1 × 10^7^ cells per DBM) scaffold as previously described [35, 36]. Before the induction of mechanical compression, NP cells were pre-cultured for 2 days under standard conditions (37 °C, 21% O_2_ and 5% CO_2_).

### Bioreactor culture and compressive loading application

**Fig. 1:**
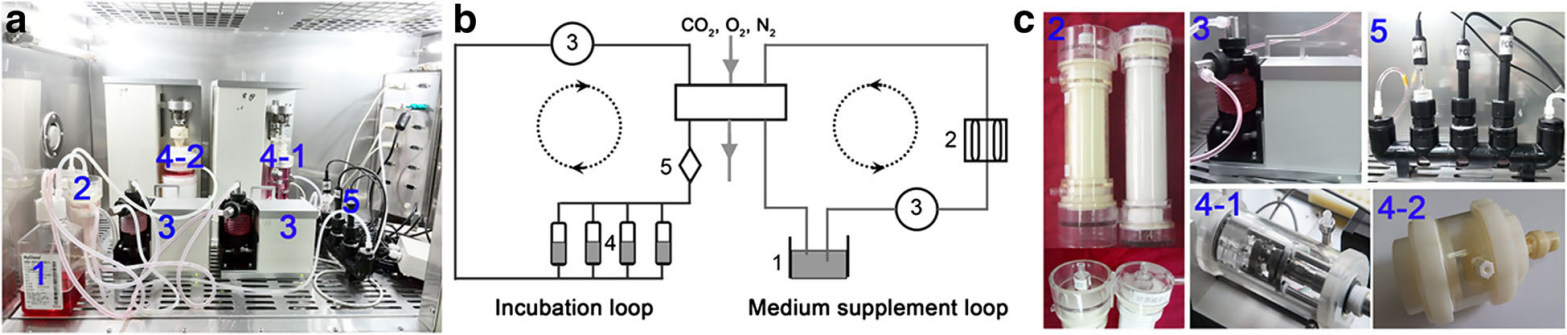
The substance exchanger-based perfusion bioreactor system. **a** Photograph of this substance exchanger-based perfusion bioreactor. **b** A schematic of this novel bioreactor system. **c** Photograph of each unit of this perfusion bioreactor (1, medium reservoir; 2, substance exchanger; 3, peristaltic pump; 4-1, the first generation of the tissue culture chamber; 4-2, the second generation of the tissue culture chamber; 5, PO2, PCO2 and pH sensors)

After 2 days of static pre-culture, NP cell-seeded scaffolds were placed into culture chambers of a substance exchanger-based perfusion bioreactor [36]. As illustrated in Fig. 1, this perfusion bioreactor mainly consists of a medium reservoir, a peristaltic pump (Jiyue Tech, Chongqing, China), a substance exchanger (Xijing Medical Co., Ltd., Xian, China), a pH sensor (PHG5202; Xuetong Instruments, Guiyang, China), a pO2 sensor (OXY5402; Xuetong Instruments), tissue culture chambers, a loading application device, and a central control system in addition to other ancillary equipment. Perfusion bioreactor culture and compressive loading application were performed for 5 days. The NP cell-seeded scaffolds were assigned to the control group (non-compression) or compression groups (2% or 20% deformation, 1.0 Hz, 6 hours per day), respectively. The compression magnitudes were defined according to the disc height alteration in one day (20–25%) and previous studies [37, 38]. To investigate the role of the p38 MAPK-ROS pathway in effects of high-magnitude compression on the premature senescence of NP cells, the specific p38 MAPK inhibitor SB203580 (10 μM, Beyotime, Haimen, China) and the ROS scavenger N-acetylcysteine (NAC, 5 mM, Beyotime) were added to the culture medium in the 20% deformation compression group, respectively. Concentrations of SB203580 and NAC were chosen according to previous studies and our own experience [39, 40].

### Cell proliferation assay

NP cells seeded in the DBM scaffold were harvested by digestion with 0.05% trypsin and 0.1% collagenase I. Then, NP cells (3 × 10^3^ cells per group) were seeded in a 96-well plate and NP cell proliferation was detected at 6, 24, and 48 hours with a Cell Counting Kit-8 (CCK-8, Beyotime). NP cell proliferation potency was also evaluated by the uptake of 5-ethynil-2’-deoxyuridine (EdU) into DNA using a Click-iT EdU microplate assay kit (Invitrogen,, Carlsbad, CA, USA) according to the manufacturer’s instructions. Briefly, after the harvested NP cells (3 × 10^3^ cells per group) were seeded in a 96-well plate, NP cells were labelled with EdU, which was coupled to Oregon Green Azide. Finally, EdU incorporated into DNA was detected with an HRP-conjugated anti-Oregon Green antibody and Amplex UltraRed. The NP cell proliferation rate was expressed in relative fluorescence units (RFU), which was detected at an excitation/emission wavelength of 490/585 nm with an automatic microplate reader (Thermo Fisher Scientific, Waltham, MA, USA).

### Intracellular ROS measurement

The intracellular ROS content was measured with a reactive oxygen species assay kit (Nanjing Jiancheng Bioengineering Institute, Nanjing, China) according to the manufacturer’s instructions. Briefly, after NP cells seeded in the DBM scaffold were incubated with the fluorescent probe DCFH-DA (10 μM) in a humidified atmosphere for 30 minutes at 37 °C, they were harvested as described above and washed with PBS twice. Finally, NP cells (1 × 10^5^ per group) were analysed on an automatic microplate reader (Thermo Fisher Scientific) and ROS generation was expressed in relative fluorescence units (RFU), which was detected at an excitation/emission wavelength of 490/585 nm.

### SA-β-Gal staining

NP cells seeded in the DBM scaffold were harvested as described above. After NP cells (1 × 10^4^ per group) were subjected to adherent culture for 8–10 hours, SA-β-Gal staining was performed according to the manufacturer’s instructions (Senescence β-Galactosidase Staining Kit, Beyotime). The SA-β-Gal stained NP cells were observed under a light microscope (Olympus BX51; Olympus, Tokyo, Japan), and then NP cell senescence, expressed as the percentage of senescent NP cells to total NP cells, was analysed with Image-Pro Plus software (Version 5.1, Media Cybernetics, Inc., Rockville, MD, USA).

### Cell cycle analysis

NP cells seeded in the DBM scaffold were harvested as described above. After rinsing with PBS and fixation with 75% ethanol overnight at 4 °C, NP cells were incubated with propidium iodide dye (50 μg/ml, Beyotime) and RNase A (100 μg/ml, Beyotime) for 30 minutes. Then, NP cells were analysed with a flow cytometry machine (FACS Aria; BD Company, Franklin Lakes, NJ, USA). The cell cycle phases (G0/G1, G2/M and S) of NP cells were analysed by multicycle software (PHENIX Company, Japan).

### Detection of cell viability

NP cell apoptosis was evaluated by flow cytometry using Annexin V/PI double staining according to the manufacturer’s instructions (Beyotime). Briefly, after NP cells were harvested as described above and washed with PBS, they were resuspended in 600 μL of Annexin V-FITC binding buffer. Then, 15 μL of Annexin V and 30 μL of PI solution were added to the NP cell suspension and incubated for 20 minutes at room temperature. Finally, the cell apoptosis rate was detected by flow cytometry (FACS Aria; BD Company) via the FACSDiva software (Becton Dickinson, Franklin Lakes, NJ, USA). These Annexin V positive-stained and PI-negative-stained cells and double positive-stained cells were regarded as dying cells in this assay.

### Telomerase activity

After NP cells seeded in the DBM scaffold were harvested as described above, NP cell pellets were incubated with RIPA lysis buffer (Beyotime) and centrifuged to collect the supernatant. Then, a telomerase (TE) enzyme-linked immunosorbent assay (ELISA) kit (Mlbio, Shanghai, China) was used to measure telomerase activity (IU/L) according to the manufacturer’s instructions.

### Real-time PCR analysis

Real-time PCR was used to analyse the gene expression of senescence markers (p16 and p53) and extracellular matrix molecules (aggrecan and collagen II). Briefly, after NP cells seeded in the DBM scaffold were harvested as described above, total RNA was extracted with Tripure Isolation Reagent (Roche, Basel, Switzerland) and further synthesized into cDNA using the First Strand cDNA Synthesis Kit (Roche). Then, real-time PCR was performed with a reaction system that contained cDNA, SYBR Green Mix (Toyobo, Osaka, Japan) and primers (Table 1). The thermal cycling for all reactions was as follows: 5 min at 95 °C, followed by 35 amplification cycles of 30 seconds at 95 °C, 20 seconds at 56 °C and 15 seconds at 72 °C. β-actin was used as an internal reference and the relative gene expression was expressed as 2^―△△Ct^.

**Table 1:** Primers of target genes

Gene	Accession number	Forward (5’-3’)	Reverse (5’-3’)
β-actin	NM_031144.3	CCGCGAGTACAACCTTCTTG	TGACCCATACCCACCATCAC
Aggrecan	XM_002723376.1	ATGGCATTGAGGACAGCGAA	GCTCGGTCAAAGTCCAGTGT
Collagen II	NM_012929.1	GCCAGGATGCCCGAAAATTAG	CCAGCCTTCTCGTCAAATCCT
P53	XM_008767773.1	CCTTAAGATCCGTGGGCGT	GCTAGCAGTTTGGGCTTTCC
P16	NM_031550.1	TACCCCGATACAGGTGATGA	TACCGCAAATACCGCACGA

### Western blotting analysis

Western blotting assay was performed to analyse the protein expression of senescence markers (p16 and p53) and p38 MAPK activity. Briefly, after total protein was extracted with the RIPA lysis solution (Beyotime) and protein concentration was measured with the BCA kit (Beyotime), protein samples were subjected to SDS-PAGE system and transferred to a PVDF membrane. After blocking with 5% bovine serum albumin, the PVDF membrane was incubated with primary antibodies (β-actin: Proteitech, Rosemont, IL, USA; 60008-1-Ig; p16: Novus, St. Louis, MO, USA; NBP2-37740; p53: Proteintch, 10442-1-AP; p38 MAPK: AM065, Beyotime; phosphor-p38 MAPK: AM063, Beyotime) at 4 °C overnight and corresponding secondary antibodies (ZSGB-BIO, Beijing, China, diluted 1:2000) at 37 °C for 2 hours. Protein bands were developed with the SuperSignal West Pico Trial Kit (Thermo Fisher Science). Protein expression was analysed with Image J software (National Institutes of Health, Bethesda, MD, USA).

### Glycosaminoglycan (GAG) content assay

The DMMB assay was performed to measure GAG synthesized by NP cells [41]. Briefly, after DBM samples were suspended in 1 mL of PBS containing 5 mg/mL papain (Sangon, Biotech Co., Ltd., Shanghai, China) for 6–8 hours at 60 °C. Then, the GAG content in the digested sample was measured at an absorbance value at 525 nm via the 1,9-dimethyl methylene blue (DMMB) assay, in which shark cartilage chondroitin sulphate (Sigma-Aldrich) was used as a standard.

### Investigation of the effects of high-magnitude compression on the premature senescence of NP cells in the rat disc organ culture

#### Isolation and organ culture of rat disc

Eighteen healthy experimental animals (Sprague-Dawley rats, male, 320–340 g, 12 weeks old) were maintained in standard housing and husbandry conditions before starting this study. The lumbar discs (L1–L5) were harvested as described in a previous study [42]. Then, the discs were cultured for 10 days in the tissue culture chamber of our self-developed bioreactor and compressed at a magnitude of 0.1 or 1.3 MP (1.0 Hz, 6 hours per day). The non-compressed discs were used as controls. The compression magnitudes used in this study were based on our previous experience and on disc pressure in daily life (0.1 MPa: lying prone; 1.3 MPa: lifting a heavy weight) [43, 44]. Fresh DMEM culture medium (Gibco) supplemented with 10% (v/v) FBS (Gibco) and 1% (v/v) penicillin-streptomycin (Gibco) was circulated at a rate of 5 mL/min (Fig. 1). Due to the discrepancy between different disc levels, discs from the same levels were used for the same assay in this study, as in a previous study [45]. For example, the immunohistochemical staining assay was performed on the same three discs (L1/2, L2/3 and L3/4) from different animals.

### Immunohistochemistry

Immunocytochemistry staining was performed on tissue sections to evaluate the protein deposition of matrix macromolecules (aggrecan and collagen II). Briefly, after discs were sequentially paraformaldehyde-fixed for 24 hours, paraffin-embedded and sectioned, the immunohistochemistry assay was performed as described previously [46]. Primary antibodies (aggrecan: Novus, NB120-11570; collagen II: Abcam, Cambridge, MA, USA; ab34712) were used at a dilution of 1:200 in this assay. Disc sections were observed under a light microscope (Olympus BX51) and analysed with Image-Pro Plus software (Version 5.1.0.20. Media Cybernetics, Inc.). The staining intensity (integral optical density, IOD) was analysed with Image-Pro Plus software (Version 5.1, Media Cybernetics, Inc.), and it was used for comparison among groups.

### Measurement of ROS content within the NP tissue

After compression, NP tissue was harvested under a dissecting microscope and NP cells were isolated as described above. Then, NP cells (1 × 10^5^ cells per group) were used to measure ROS generation as described above.

### Real-time PCR assay and western blotting assay

After compression, NP tissue was harvested as described above. Then, as described above, real-time PCR was performed to analyse the gene expression of senescence markers (p16 and p53) and matrix molecules (aggrecan and collagen II), and western blotting was performed to analyse senescence marker protein expression (p16 and p53), matrix molecule protein expression (aggrecan: Santa Cruz Biotechnology, Dallas, TX, USA; sc-16492; collagen II: Abcam, ab34712) and p38 MAPK activity.

### GAG content measurement

After the wet weight was recorded, the isolated NP samples were thoroughly digested with 1 mL of papain solution overnight. Then, GAG content (normalized to the wet tissue weight) was determined via the dimethyl methylene blue (DMMB) assay as described above.

### Statistical analysis

All data are expressed as the means ± SD, and each experiment was performed in triplicate in this study. After the homogeneity test for variance, comparisons between groups were performed via one-way analysis of variance (ANOVA) using SPSS 13.0 software (SPSS Inc., Chicago, IL, USA) and the post hoc test was determined by the LSD test. A significant difference was indicated when the *p* value < 0.05. Although the statistical processing was performed according to the standard procedures, our limited sample size may have introduced some inaccuracy to the statistical results.

## Results

### Experiments performed on the NP cell scaffold culture

#### High-magnitude compression induced ROS generation in NP cells

**Fig. 2:**
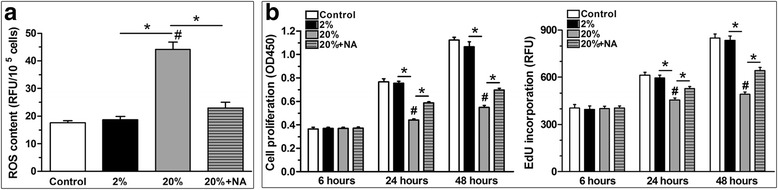
Measurement of reactive oxygen species (ROS) generation and cell proliferation of nucleus pulposus (NP) cells from scaffold culture. **a** 20% deformation compression increased ROS generation, which was reversed by treatment with the ROS scavenger NAC. **b** NP cells from the 20% deformation group had a decreased cell proliferation potency compared with those from the 2% deformation group. Data are expressed as the mean ± SD (*n* = 3). *Indicates a significant difference (*p* < 0.05) between two groups; #indicates a significant difference (*p* < 0.05) compared with the control group

Results showed that 20% deformation compression significantly increased ROS generation compared with 2% deformation compression. As expected, ROS generation in the 20% deformation group was inhibited when the ROS scavenger NAC was added to the culture medium (Fig. 2a).

### High-magnitude compression inhibited NP cell proliferation and decreased cell viability, which was partly reversed by treatment with the ROS scavenger NAC

Senescent cells often have limited cell proliferation potency. The CCK-8 and EdU incorporation assay showed that NP cell proliferation potency in the 20% deformation group decreased compared with that in the 2% deformation group at 24 and 48 hours. However, treatment with the ROS scavenger NAC slightly increased NP cell proliferation in the 20% deformation group (Fig. 2b). To investigate cell viability under mechanical compression, we evaluated NP cell viability via flow cytometry. The results showed that the percentage of dying NP cells in the 20% deformation compression group (22.17%) increased compared with the 2% deformation compression group (4.31%) and the control group (3.55%). However, treatment with the ROS scavenger NAC decreased the percentage of dying NP cells in the 20% deformation compression group (from 22.17% to 16.83%. See Additional file 2: Figure S2).

### High-magnitude compression promoted NP cell senescence and this effect was alleviated by the ROS scavenger NAC

**Fig. 3:**
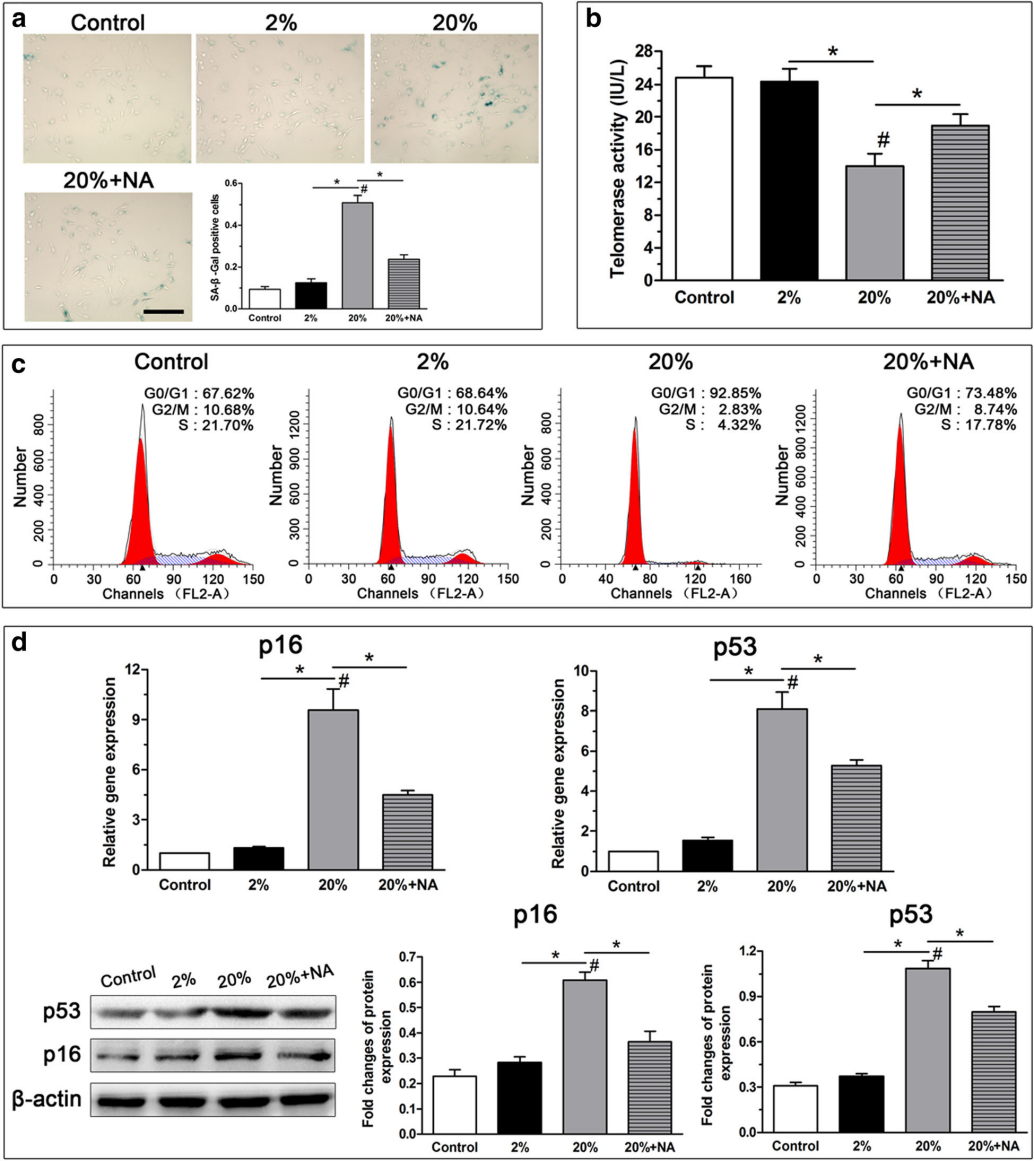
Analysis of SA-β-Gal activity, telomerase activity, G1 cell cycle arrest and senescence marker (p16 and p53) expression in the nucleus pulposus (NP) cells from scaffold culture. 20% deformation compression significantly increased SA-β-Gal activity (**a**), decreased telomerase activity (**b**), promoted G1 cell cycle arrest (**c**) and upregulated senescence markers expression (**d**). The addition of the ROS scavenger NAC attenuated these effects caused by 20% deformation compression. Scale = 100 μm. Data are expressed as the mean ± SD (*n* = 3). *Indicates a significant difference (*p* < 0.05) between two groups; #indicates a significant difference (*p* < 0.05) compared with the control group

In this study, we investigated the effects of compression on parameters of cellular senescence. The results showed that 20% deformation compression significantly promoted NP cell senescence, as reflected by increased SA-β-Gal activity (Fig. 3a), decreased telomerase activity (Fig. 3b), aggravated G1 cell cycle arrest (Fig. 3c) and increased expression of senescence markers (p16 and p53) (Fig. 3d). However, treatment with the ROS scavenger NAC markedly attenuated NP cell senescence in the 20% deformation group.

### High-magnitude compression attenuated matrix anabolism of NP cells, and this effect was partly inhibited by treatment with the ROS scavenger NAC

**Fig. 4:**
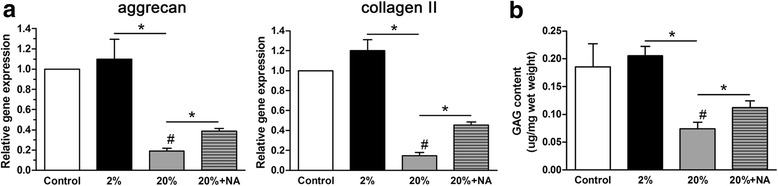
Gene expression of matrix macromolecules (aggrecan and collagen II) and glycosaminoglycan (GAG) content from nucleus pulposus (NP) cells in scaffold culture. 20% deformation compression significantly downregulated gene expression of matrix molecules (**a**) and decreased GAG content (**b**), which could be partly reversed by treatment with the ROS scavenger NAC. Data are expressed as the mean ± SD (*n* = 3). *Indicates a significant difference (*p* < 0.05) between two groups; #indicates a significant difference (*p* < 0.05) compared with the control group

Because senescent cells are often hypoactive in terms of matrix synthesis [47], we examined the gene expression of matrix macromolecules (aggrecan and collagen II) and GAG content in NP cells. As shown in Fig. 4, 20% deformation compression significantly decreased the gene expression of matrix macromolecules (aggrecan and collagen II) and GAG content in NP cells compared with 2% deformation compression. However, treatment with the ROS scavenger NAC could markedly upregulate the expression of matrix macromolecules (aggrecan and collagen II) and increased the GAG content in NP cells in the 20% deformation group (Fig. 4).

### Increased phosphorylation of p38 MAPK in NP cells under high-magnitude compression was not attenuated by treatment with the ROS scavenger NAC

**Fig. 5:**
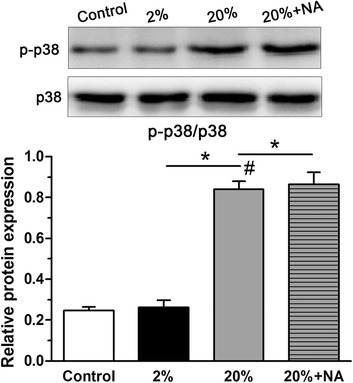
Analysis of p38 MAPK activity from nucleus pulposus (NP) cells in scaffold culture. 20% deformation compression significantly increased p38 MAPK activity and treatment with the ROS scavenger NAC had no significant effect on 20% deformation compression-induced p38 MAPK pathway activation. Data are expressed as the mean ± SD (*n* = 3). *Indicates a significant difference (*p* < 0.05) between two groups; #indicates a significant difference (*p* < 0.05) compared with the control group

The p38 MAPK pathway can be activated under mechanical stimulation [39]. In this study, the expression of phospho-p38 MAPK was increased in the 20% deformation group compared with that in the 2% deformation group. However, the ROS scavenger had no significant effect on the expression of phospho-p38 MAPK (Fig. 5).

### p38 MAPK inhibition decreased ROS generation, promoted NP cell proliferation and increased NP cell viability under high-magnitude compression

**Fig. 6:**
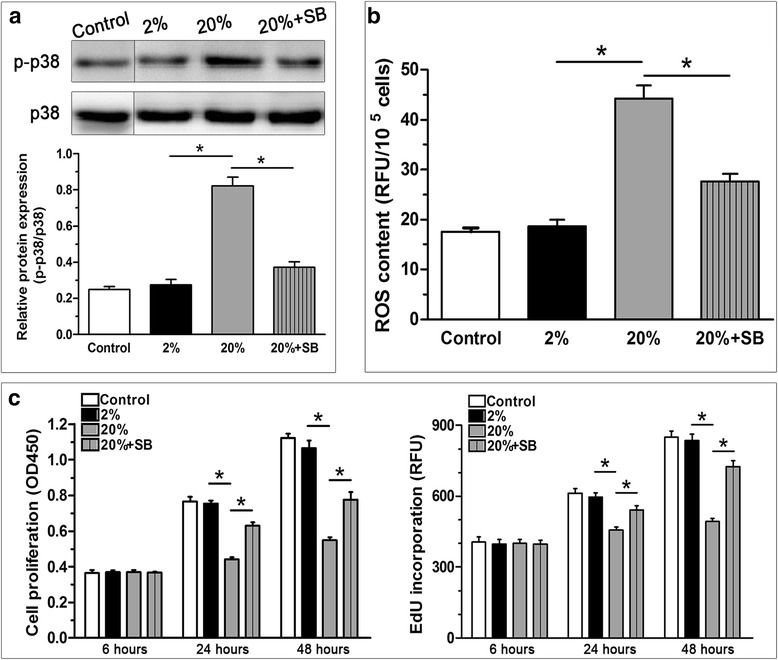
Effects of p38 MAPK inhibition on reactive oxygen species (ROS generation and the cell proliferation of nucleus pulposus (NP) cells in the 20% deformation compression group. The specific inhibitor SB203580 significantly inhibited p38 MAPK pathway activation (**a**). Inhibition of the p38 MAPK pathway decreased ROS generation (**b**) and suppressed the proliferation potency (**c**) of NP cells in the 20% deformation compression group. Data are expressed as the mean ± SD (*n* = 3). *Indicates a significant difference (*p* < 0.05) between the two groups. RFU relative fluorescence units

To detect the relationship between the p38 MAPK pathway and ROS generation, ROS generation in NP cells compressed at 20% deformation was measured after the addition of SB203580. As shown in Fig. 6, the addition of SB203580 effectively inhibited p38 MAPK activity (Fig. 6a) and significantly decreased ROS generation (Fig. 6b) in NP cells in the 20% deformation compression group. Additionally, the inhibition of the p38 MAPK pathway partly reversed high-magnitude compression-induced NP cell proliferation suppression at 24 and 48 hours (Fig. 6c). Moreover, the percentage of dying NP cells in the 20% deformation group was significantly decreased by the inhibitor SB203580 (from 22.17% to 11.65%. See Additional file 2: Figure S2).

### p38 MAPK inhibition significantly decreased ROS generation, attenuated cell senescence and promoted matrix anabolism under high-magnitude compression

**Fig. 7:**
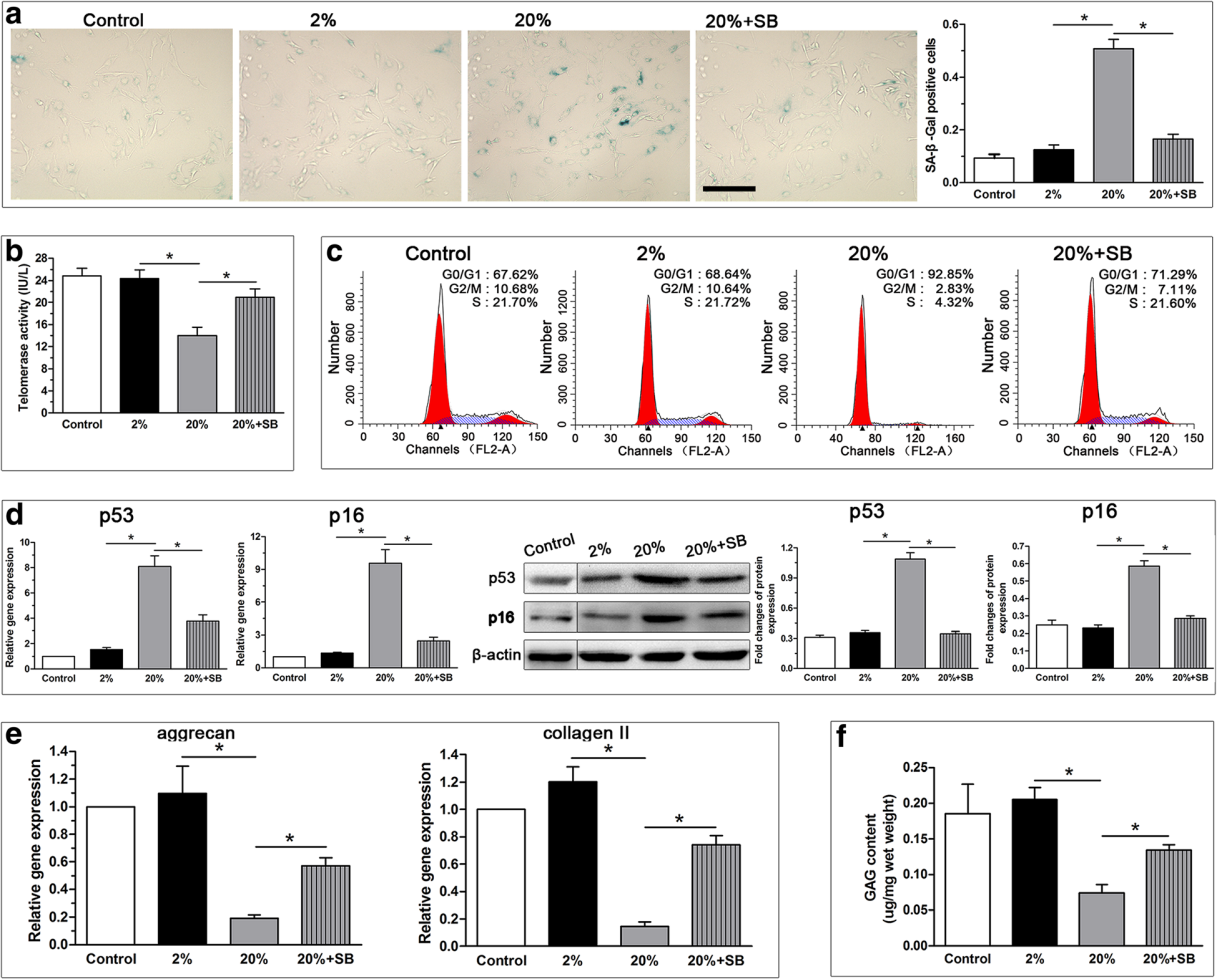
Effects of p38 MAPK inhibition on senescence associated β-galactosidase (SA-β-Gal) activity, telomerase activity, G1 cell cycle arrest and senescence marker (p16 and p53) expression, matrix molecule (aggrecan and collagen II) expression and glycosaminoglycan (GAG) content of nucleus pulposus (NP) cells in the 20% deformation compression group. The results showed that p38 MAPK inhibition decreased SA-β-Gal activity (**a**), increased telomerase activity (**b**), attenuated G1 cell cycle arrest (**c**), downregulated senescence marker (p16 and p53) expression (**d**), upregulated matrix molecule (aggrecan and collagen II) expression (**e**) and increased glycosaminoglycan (GAG) content (**f**) of nucleus pulposus (NP) cells in the 20% deformation compression group. Data are expressed as the mean ± SD (*n* = 3). *Indicates a significant difference (*p* < 0.05) between the two groups

We also investigated the role of p38 MAPK pathway in high-magnitude compression-induced NP cell senescence. The results showed that the addition of SB203580 partly abolished NP cell senescence induced by high (20% deformation) compression, which was indicated by the decreased SA-β-Gal activity (Fig. 7a), increased telomerase activity (Fig. 7b), attenuated G1 cell cycle arrest (Fig. 7c), and decreased senescence marker (p16 and p53) expression (Fig. 7d). Additionally, SB203580 upregulated the gene expression of matrix molecules (aggrecan and collagen II) (Fig. 7e) and increased the GAG content (Fig. 7f) of NP cells in the 20% deformation compression group.

### Experiments performed on the ex vivo disc organ culture

**Fig. 8:**
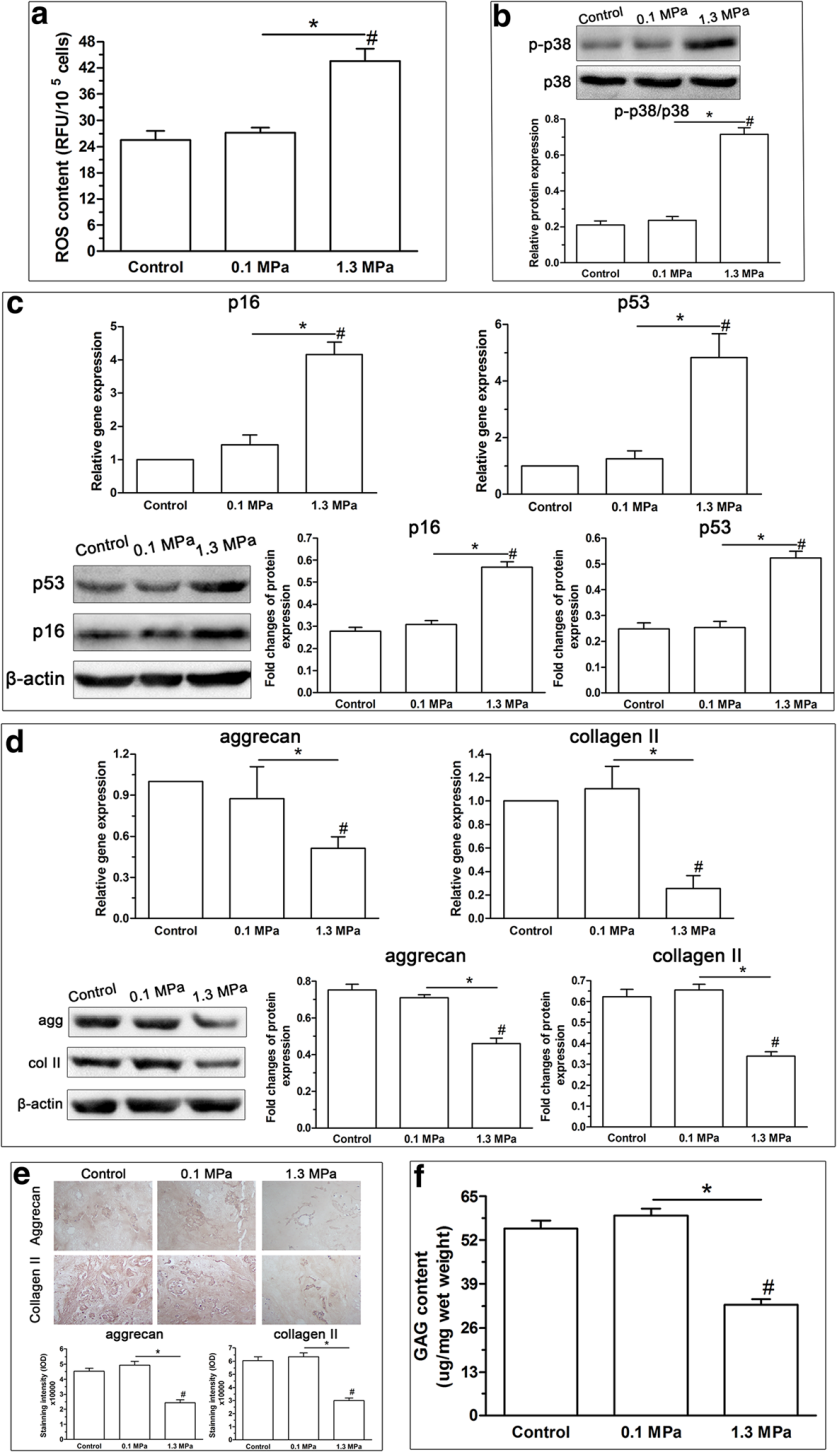
Effects of high compression on reactive oxygen species (ROS) generation, p38 MAPK activity, senescence phenotype and matrix homeostatic phenotype of nucleus pulposus (NP) cells in rat disc organ culture. The results showed that high compression (1.3 MPa) increased ROS generation (**a**), upregulated p38 MAPK activation (**b**), promoted the senescence phenotype (**c**), and inhibited matrix homeostatic phenotype (**d**-**f**) of NP cells compared with low compression (0.1 MPa). Data are expressed as the mean ± SD (*n* = 3). *Indicates a significant difference (*p* < 0.05) between the two groups. GAG glycosaminoglycan, RFU relative fluorescence units

To further verify the effects of high-magnitude compression on NP cell senescence, we applied dynamic compression to organ-cultured rat discs. The results showed that high-magnitude compression (1.3 MPa) significantly promoted the NP cell senescence phenotype compared with low compression (0.1 MPa), indicated by increased senescent marker (p16 and p53) expression (Fig. 8c), downregulated matrix molecule (aggrecan and collagen II) expression (Fig. 8d) and decreased matrix protein deposition (Fig. 8e) and GAG content (Fig. 8f) within the NP tissue compared with the low compression (0.1 MPa) group. In addition, ROS generation and p38 MAPK activity were also increased in the high-magnitude compression (1.3 MPa) group compared with the low compression (0.1 MPa) group (Fig. 7a, b).

## Discussion

Cellular senescence within the degenerative disc NP tissue is a common pathological phenomenon [5, 6]. ROS-induced oxidative stress is able to promote cellular senescence in many cells [17]. Under physiological situations, the disc is subjected to various types of mechanical stimuli; dynamic compression is a typical loading form [48]. In cartilage explants, excessive mechanical compression can cause increased ROS generation [26]. However, whether high-magnitude compression can lead to NP cell senescence and elevate ROS generation in NP cells is not completely understood. To our knowledge, this is the first study to report the effects of compression on NP cell senescence. In the present study, we hypothesized that high-magnitude compression can accelerate NP cell senescence through the p38 MAPK/ROS pathway. To examine this hypothesis, NP cell scaffold-culture and intact disc organ culture were used to analyse the effects of compression on NP cell senescence and the role of the p38 MAPK/ROS pathway in this regulatory process.

In this study, high-magnitude compression accelerated NP cell senescence in NP cell scaffold culture and disc organ culture, which is shown by increased SA-β-Gal activity, upregulated senescence marker (p16 and p53) expression, increased G1 cell cycle arrest, decreased cell proliferation potency, telomerase activity and downregulated matrix macromolecule (aggrecan and collagen II) expression. Additionally, high-magnitude compression promoted intracellular ROS generation in NP cell scaffold culture and disc organ culture, which is also documented in studies performed on cartilage explants [26]. However, treatment with the ROS scavenger NAC partly inhibited NP cell senescence induced by high-magnitude compression. Together, these results demonstrate that high-magnitude compression can lead to NP cell senescence by increasing ROS generation. This is a novel mechanism for mechanical stimuli-induced disc degeneration. Another important finding is that 20% deformation compression increased the percentage of dying NP cells compared with 2% deformation compression. This is in line with most previous studies that reported the effects of mechanical load on disc cell biology. This difference in cell viability may partly contribute to differences in cell proliferation results. However, the high-magnitude (20% deformation) compression also caused other senescence-associated phenotypes. Therefore, we think that high-magnitude compression can simultaneously promote NP cell death and NP cell senescence.

It is well established that senescent cells often display an attenuated synthetic ability and transform to a catabolic metabolism [47]. In this study, we also found that senescent NP cells induced by high-magnitude compression had decreased matrix (aggrecan and collagen II) deposition and a downregulated matrix gene expression in scaffold culture. Excessive compressive loading is regarded as a negative external factor for disc biology [48]. In line with this opinion, our previous study also demonstrated that a high compressive magnitude induced degenerative changes within the disc tissue, such as decreased matrix biochemical content, upregulated matrix degrading enzymes and downregulated matrix genes and tissue inhibitors of matrix metalloproteinase [42, 46, 49]. Together, the attenuated matrix homeostatic phenotype of NP cells under high-magnitude compression also indirectly suggests that high-magnitude compression promotes NP cell senescence.

In the scaffold culture, the intracellular ROS content in the high-magnitude compression (20% deformation) group was higher than that in the low compression (2% deformation) group. This is in line with previous studies that demonstrated that excessive loading increased ROS generation in cartilage tissue [26]. Appropriate ROS concentration functions as the intracellular messenger that can offer a chemical link between structural changes and functions of transcription factors [50]. However, excessive ROS generation is toxic to cells because it can cause a loss of cell function, cell cycle arrest and cell apoptosis [51]. Consistent with the above statement, cellular senescence was positively correlated with ROS generation in NP cells subjected to high mechanical compression (both in the scaffold culture and disc organ culture). Moreover, the inhibition of ROS generation in the NP cells subjected to high-magnitude compression partly attenuated NP cell senescence in scaffold culture. This further indicates that increased ROS generation can lead to cellular senescence in NP cells.

The p38 MAPK pathway, a typical subclass of MAPKs, converts extracellular stimuli into cellular biological responses to regulate cell bioactivities that include apoptosis, proliferation and senescence [28]. Previous studies on chondrocytes revealed that the p38 MAPK signalling pathway can be activated by mechanical stimuli [29–31]. In addition, activation of the p38 MAPK pathway can regulate the mechanical stimulation-induced apoptosis of endplate chondrocytes in organ-cultured mouse discs [39]. In the present study, the p38 MAPK pathway was also activated under dynamic compression in NP cell scaffold culture. Furthermore, inhibition of the p38 MAPK pathway partly counteracted the cellular senescence of dynamically compressed NP cells. This indicates that p38 MAPK is involved in the effects of dynamic compression on NP cell senescence. On the other hand, when we treated NP cells with the ROS inhibitor NAC in the 20% deformation group, we found that NAC could significantly decrease ROS generation and subsequent NP cell senescence in the 20% deformation group and that p38 activity was not attenuated by NAC and was even increased after NAC addition in the 20% deformation group (Fig. 5). However, inhibition of the p38 MAPK pathway can partly decrease ROS generation and NP cell senescence in the 20% deformation group (Figs. 6 and 7). Therefore, in this study, it appears that p38 MAPK is the upstream effecter of ROS and regulates, at least in part, high-magnitude compression-induced NP cell senescence through ROS generation.

Compared with the NP cell scaffold culture, disc organ culture is more advantageous for the study of NP cell biology because it maintains the natural cell-cell and cell-matrix interaction [52, 53]. In addition, the bioreactor platform is useful, as it more closely resembles the physiological condition [54]. Therefore, to verify the effects of compression on NP cell senescence, we performed similar experiments on the intact rat disc bioreactor culture. Consistent with the NP cell scaffold culture, high-magnitude compression (1.3 MPa) could also increase ROS generation and promoted the cellular senescence of NP cells in the disc organ culture when compared with low compression (0.1 MPa). These findings further indicate that high-magnitude compression can promote NP cell senescence, and that high-magnitude compression can increase p38 MAPK activity and ROS generation.

This study also has some limitations. First, NP cells were scaffold-cultured under normoxic conditions that differ from the physiological conditions in which NP cells are contained in a three-dimensional (3D) environment under hypoxic conditions. Second, two sources of ROS generation exist: endogenic and ectogenic. These two types of ROS may cause different signals in cell biology. However, the ROS generated by mechanical stress in this study is mainly endogenic. Further studies are needed to verify whether the ectogenic ROS under high-magnitude compression has similar effects on NP cell senescence. Third, although we verified the positive effects of high-magnitude compression on NP cell senescence in the rat disc organ culture, the roles of the p38 MAPK pathway and ROS generation in this process were not further investigated using their inhibitors. However, our previous study has demonstrated that inhibition of the p38 MAPK pathway attenuates high-magnitude compression-induced NP cell senescence in the porcine disc organ culture [55]. Additionally, previous studies have demonstrated that inhibition of ROS inhibits disc cell senescence and ageing-related disc degeneration in vitro and in vivo [22, 56, 57]. Therefore, we speculate that either inhibition of the p38 MAPK pathway or suppression of ROS generation can attenuate high-magnitude compression-induced NP cell senescence in the rat disc organ culture.

## Conclusions

Based on our results, we can conclude that high-magnitude compression can promote NP cell senescence, and the p38 MAPK-ROS pathway is involved in this regulatory process. This study, for the first time, sheds light on the effects of compression on disc NP cell senescence and will ultimately contribute to an understanding of the mechanism that underlies the advanced disc degeneration induced by excessive mechanical loading.

## Additional files

Additional file 1: Figure S1. Identification of nucleus pulposus (NP) cells. (A) NP cells under a light microscope. NP cells exhibited short shuttle-like, round or polygon shapes. Magnification: ×200. (B) Analysis of gene expression of NP cell-specific markers (CAXII, Keratin-19, FOXF1, and PAX1). (PDF 476 kb)

Additional file 2: Figure S2. Analysis of the percentage of dying nucleus pulposus (NP) cells in each group. The flow cytometry assay showed that the percentage of dying NP cells in the 20% deformation compression group (22.17%) increased compared with the 2% deformation compression group (4.31%) and the control group (3.55%). However, treatment with the ROS scavenger NAC and the p38 MAPK inhibitor SB203580 decreased 20% deformation compression-induced NP cell apoptosis (from 22.17 to 16.83% and from 22.17 to 11.65%, respectively). (PDF 353 kb)

## Abbreviations

3D: Three-dimensional
ANOVA: One-way analysis of variance
CCK-8: Cell Counting Kit-8
DBM: Decalcified bone matrix
DMMB: 1,9-dimethyl methylene blue
EdU: 5-ethynil-2’-deoxyuridine
ELISA: Enzyme-linked immunosorbent assay
FBS: Fetal bovine serum
GAG: Glycosaminoglycan
IDD: Intervertebral disc degeneration
IOD: Integral optical density
IVD: Intervertebral disc
MAPKs: Mitogen-activated protein kinases
NAC: N-acetylcysteine
NP: Nucleus pulposus
RFU: Relative fluorescence units
ROS: Reactive oxygen species
SA-β-Gal: Senescence associated β-galactosidase
