# Supplementary material for: High-magnitude compression accelerates the premature senescence of nucleus pulposus cells via the p38 MAPK-ROS pathway
Source: Arthritis Res Ther. 2017 Sep 18;19:209. doi: 10.1186/s13075-017-1384-z (PMC5604423; doi:10.1186/s13075-017-1384-z)

Additional file 1. Identification of nucleus pulposus (NP) cells. A: NP cells under a light microscope. NP cells exhibited short shuttle-like, round or polygon shapes. Magnification: 200x. B: Analysis of gene expression of NP cell specific markers (CAXII, Keratin-19, FOXF1 and PAX1).

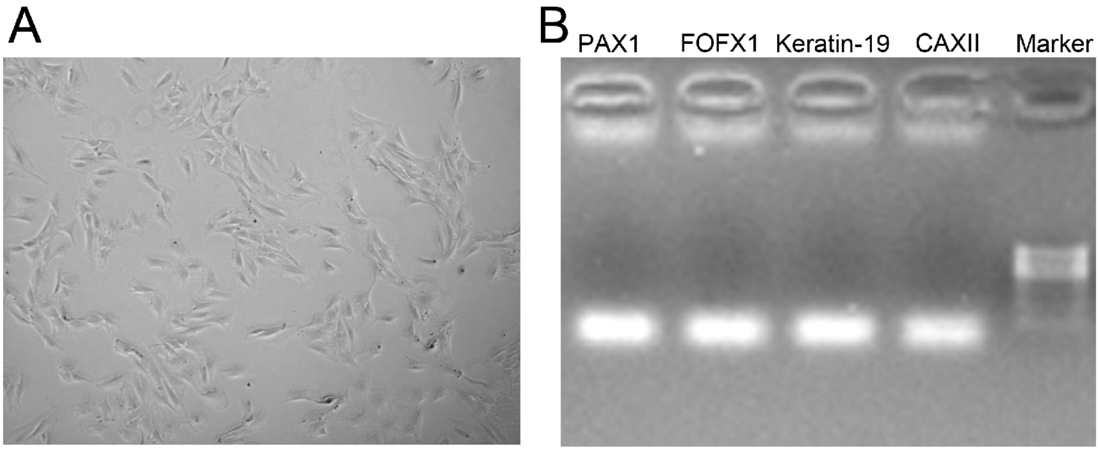

Supplement: Supplementary file 1 — Identification of nucleus pulposus (NP) cells. (A) NP cells under a light microscope. NP cells exhibited short shuttle-like, round or polygon shapes. Magnification: ×200. (B) Analysis of gene expression of NP cell-specific markers (CAXII, Keratin-19, FOXF1, and PAX1). (PDF 476 kb) [file 13075_2017_1384_MOESM1_ESM.pdf]
