# Supplementary material for: High-magnitude compression accelerates the premature senescence of nucleus pulposus cells via the p38 MAPK-ROS pathway
Source: Arthritis Res Ther. 2017 Sep 18;19:209. doi: 10.1186/s13075-017-1384-z (PMC5604423; doi:10.1186/s13075-017-1384-z)

**Additional file 2.** Analysis of the percentage of dying nucleus pulposus (NP) cells in each group. The flow cytometry assay showed that the percentage of dying NP cells in the 20% deformation compression group (22.17%) increased compared with the 2% deformation compression group (4.31%) and the control group (3.55%). However, treatment with the ROS scavenger NAC and the p38 MAPK inhibitor SB203580 decreased 20% deformation compression-induced NP cell apoptosisp (from 22.17% to 16.83% and from 22.17% to 11.65%, respectively).

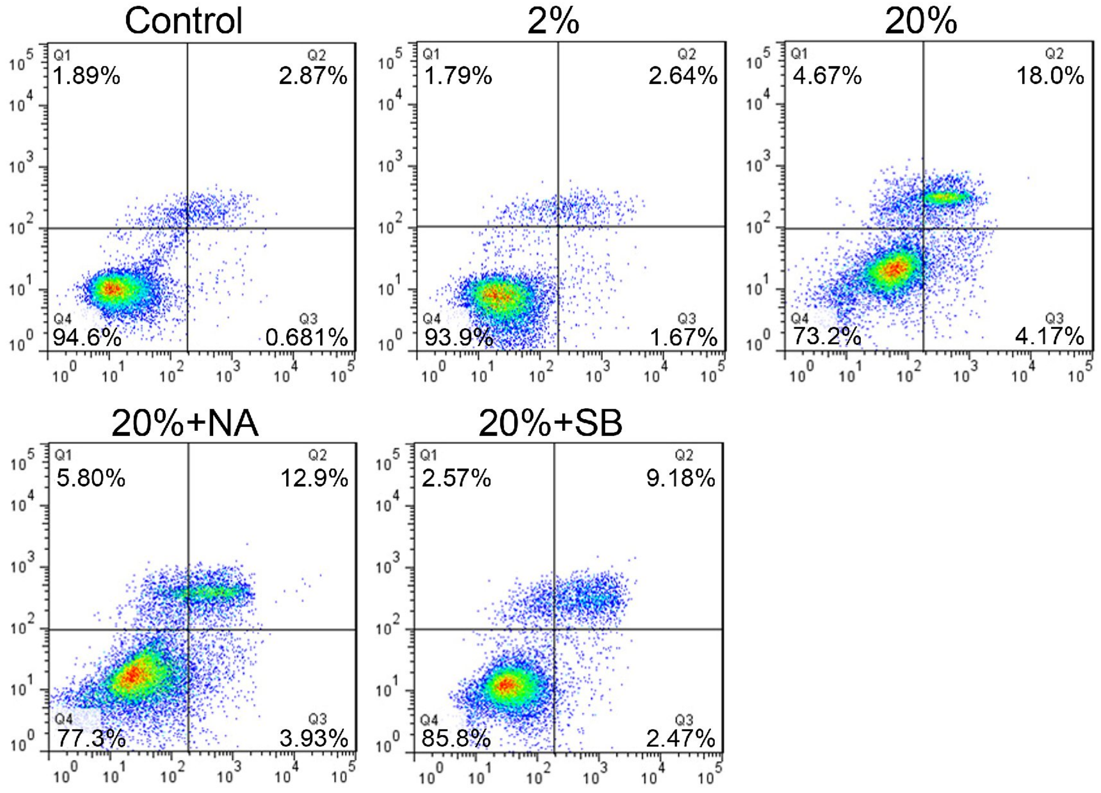

Supplement: Supplementary file 2 — Analysis of the percentage of dying nucleus pulposus (NP) cells in each group. The flow cytometry assay showed that the percentage of dying NP cells in the 20% deformation compression group (22.17%) increased compared with the 2% deformation compression group (4.31%) and the control group (3.55%). However, treatment with the ROS scavenger NAC and the p38 MAPK inhibitor SB203580 decreased 20% deformation compression-induced NP cell apoptosis (from 22.17 to 16.83% and from 22.17 to 11.65%, respectively). (PDF 353 kb) [file 13075_2017_1384_MOESM2_ESM.pdf]
